# Age and ocular toxoplasmosis: a narrative review

**DOI:** 10.1093/femsmc/xtaf002

**Published:** 2025-02-26

**Authors:** Alejandra de-la-Torre, Germán Mejía-Salgado, Armin Taghavi Eraghi, Uwe Pleyer

**Affiliations:** Neuroscience Research Group (NEUROS), Neurovitae Center for Neuroscience, Institute of Translational Medicine (IMT), Escuela de Medicina y Ciencias de la Salud, Universidad del Rosario, Bogotá, Colombia; Ophthalmology Interest Group Universidad del Rosario. (OIG UR). Escuela de Medicina y Ciencias de la Salud, Universidad del Rosario, Bogotá, Colombia; Neuroscience Research Group (NEUROS), Neurovitae Center for Neuroscience, Institute of Translational Medicine (IMT), Escuela de Medicina y Ciencias de la Salud, Universidad del Rosario, Bogotá, Colombia; Ophthalmology Interest Group Universidad del Rosario. (OIG UR). Escuela de Medicina y Ciencias de la Salud, Universidad del Rosario, Bogotá, Colombia; Health Sciences Faculty, Universidad Autónoma de Bucaramanga UNAB, Bucaramanga, Colombia; Health Sciences Faculty, Universidad Autónoma de Bucaramanga UNAB, Bucaramanga, Colombia; Health Sciences Faculty, Universidad Autónoma de Bucaramanga UNAB, Bucaramanga, Colombia; Department of Ophthalmology, Charité Campus Virchow Klinikum, Universitätsmedizin Berlin, Berlin, Germany; Berlin Institute of Health, Berlin, Germany

**Keywords:** age, ocular, toxoplasmosis, narrative, review, risc factors

## Abstract

*Toxoplasma gondii* is an extremely “successful” opportunistic parasite that infects most warm-blooded animals, including humans. While the infection is generally largely asymptomatic, the infection of the eye presenting as ocular toxoplasmosis (OT) is a potentially blinding consequence. OT remains the most common cause of infectious retinochoroiditis and places a considerable socio-economic burden on societies, particularly in developing countries. Age is one of several factors influencing the clinical presentation and outcomes of OT. Older patients often exhibit more severe disease manifestations, larger retinal lesions, and poorer visual outcomes compared to younger individuals. This disparity is attributed to immunosenescence, the age-related decline in immune function, which impairs the body's ability to control the infection effectively. Consequently, older individuals are at a higher risk of severe complications and recurrent episodes of inflammation. Understanding the interplay between age and immune response is essential for developing targeted treatment strategies and improving patient outcomes in OT.

## Teaser

Age is a critical determinant in the pathogenesis, clinical features, and outcomes of ocular Toxoplasmosis, with different age groups exhibiting distinct immunological responses and disease manifestations.

## Introduction

*Toxoplasma gondii* is a versatile intracellular parasite that infects most warm-blooded animals, including humans. The parasite has a complex life cycle involving two main stages: tachyzoites, which rapidly multiply and spread the infection, and bradyzoites, which form tissue cysts and remain dormant (Pleyer et al. 2014). Felines are the definitive host where the parasite undergoes sexual reproduction. *Toxoplasma gondii* infection can occur by ingesting food or water contaminated with cysts or oocysts. Medically, toxoplasmosis remains often asymptomatic in healthy individuals but can cause flu-like symptoms such as fever, swollen lymph nodes, and muscle pain. Notable, ocular involvement is an important consequence of *T. gondii* infection in 2%–18% of cases, resulting in irreversible retinochoroidal scars, often recurrent episodes and complications that can threaten vision (Goh et al. 2023). The clinical presentation and course of ocular toxoplasmosis (OT) vary widely, influenced by several factors, including the host’s immune status, genetic background, and age (Naranjo-Galvis et al. 2018, Mantilla-Muriel et al. 2020, De Angelis et al. 2021, Kalogeropoulos et al. 2022, 2023, Eraghi et al. 2024).

Age is a critical determinant in the pathogenesis, clinical features, and outcomes of OT, with different age groups exhibiting distinct immunological responses and disease manifestations​ (Garweg et al. 2008, De Angelis et al. 2021, Eraghi et al. 2024). The immune system undergoes significant changes with aging, a process termed immunosenescence, characterized by a decline in innate and adaptive immune functions​ (Weiskopf et al. 2009). This decline is associated with increased susceptibility to infections, including *T. gondii*, and altered disease outcomes in older individuals​ (Weiskopf et al. 2009, Eraghi et al. 2024). In younger patients, macular involvement is more common (Mets et al. 1996). Conversely, the attenuated immune response due to immunosenescence in older adults compared to middle-aged adults may result in more extensive lesions and a lower antibody index (Eraghi et al. 2024).

This review aims to explore the influence of age on epidemiology, the risk of ocular involvement, clinical characteristics, and recurrences in OT. Understanding how age affects the disease course in OT is crucial for optimizing management strategies and improving patient outcomes across different age groups.

## Influence of age on the epidemiology of ocular toxoplasmosis

The impact of age on the prevalence of OT is profound and multifaceted, reflecting differences between congenital and postnatally acquired infections. Among individuals with congenital *T. gondii* infections, the risk of developing ocular disease is remarkably high. Historical data suggest that up to 85% of children born with congenital toxoplasmosis eventually develop ocular involvement, even when the infection is subclinical at birth (Couvreur and Desmonts 1962, Stagno et al. 1977, Wilson et al. 1980). This elevated risk is primarily attributed to the underdeveloped immune system of the fetus, which is insufficiently equipped to control the spread of the parasite, thereby allowing for more extensive retinal involvement (Koppe et al. 1986). Moreover, studies indicate that ocular lesions may not manifest until several years after birth, likely due to the reactivation of quiescent tissue cysts within the retina (Koppe et al. 1986).

In contrast, the prevalence of OT among individuals who acquire *T. gondii* infections postnatally is considerably lower in some geographic areas. Epidemiological data from the USA, derived from the National Health and Nutrition Examination Survey (NHANES) surveys, indicate that ~2% of seropositive individuals develop ocular lesions, which is lower than the rates observed in congenital cases (Holland 2003). However, in regions such as Southern Brazil, where most *T. gondii* infections are acquired postnatally, ocular involvement can be as high as 18% (Silveira et al. 1988, Glasner et al. 1992). Similarly, in a study conducted in Colombia, a prevalence of 10.6% of retinochoroidal scars due to *T. gondii* (17/161) was found in individuals without a previous diagnosis (Gómez-Marín et al. 2021). The disparity in prevalence rates between geographic regions has been partially attributed to more virulent parasite strains in these areas (de-la-Torre et al. 2013, Pfaff et al. 2014) and differences in the age at which individuals are initially exposed to the parasite (De Moura et al. 2006). For example, in Southern Brazil, infections tend to be acquired during childhood, with seroprevalence reaching 85% by adolescence (Silveira et al. 2001). Moreover, in Colombia, a 60% positivity in at least one sample of food–water in public schools was found, and 101/311 (33%) children were IgG positive (Luna et al. 2019), which is typically different from North America, where the prevalence in childhood is lower (10.9%) (Van Den Berg et al. 2023).

Therefore, the cumulative prevalence of OT within a population is heavily influenced by the relative proportions of congenital vs. postnatally acquired infections. The overall prevalence of ocular disease tends to increase with age, reflecting the gradual accumulation of new infections years after transmission and recurrences. Data from NHANES III (1988–1994) revealed an age-adjusted seroprevalence of *T. gondii* IgG antibodies at 22.5% in the USA, with a clear trend of increasing prevalence with age (Jones 2001). More recent data from NHANES (2011–2014) further support this trend, showing that the seroprevalence of *T. gondii* continues to increase with age, with older adults having a significantly higher likelihood of being seropositive compared to younger individuals (53.7 vs. 43.6 years, *P* < 0.001)​ (Huang et al. 2022). This pattern is consistent with findings from European countries, such as the Netherlands and France, where seroprevalence also increases with age (Papoz et al. 1986, Carme and Tirard-Fleury 1996, Van Den Berg et al. 2023). For example, in the Netherlands, the third nationally representative serosurvey on *T. gondii* seroprevalence conducted between 2016 and 2017 found an increase from 10.9% in children and adolescents aged 0–19 years to 36.7% in adults aged 20–89 years (Van Den Berg et al. 2023), indicating a constant risk of infection across different age groups, with higher prevalence observed in older populations (Papoz et al. 1986, Carme and Tirard-Fleury 1996, Van Den Berg et al. 2023).

More recent data extrapolated from Healthcare Claims Data in Germany indicate an incidence of 9.6/100 000 toxoplasmosis patients in the general population. The *T. gondii* seroprevalence was again strongly age-associated and ranged from 20% among patients 18–29 to 77% in the older population between 70–79 years of age (Krings et al. 2021).

In some regions like Brazil, Colombia, and Panama, seroprevalence rises rapidly during the early years of life in regions. In Southern Brazil, for example, the prevalence of OT increases from 0.9% in 1 to 8-year-olds to 4.3% in 9 to 12-year-olds and 21.3% between 13- to 16-year-olds (Silveira et al. 1988, Glasner et al. 1992). Likewise, in Colombia, a serological prevalence of *T gondii* has been reported at 43.2% (41/95) in healthy children (<18 years) (Ek et al. 2012). This rapid increase in seroprevalence during childhood has also been observed in Germany, where Giese et al. documented a *T. gondii* seroprevalence of 6.3% in female children and adolescents. With each additional year of life, the likelihood of being seropositive increased by 1.2, indicating a strong force of infection. Social status and municipality size were associated with seropositivity (Giese et al. 2024). In contrast, studies from the Netherlands have reported a more gradual increase in seroprevalence, with a seroprevalence of 10.9% among children and adolescents aged 0–19 years (Van Den Berg et al. 2023). Reflecting different regional patterns. The seroprevalence of *T. gondii* IgG antibodies is rising in an almost linear order (~1% per year) from around 20% in teens and young adults (age 18–29) to 77% in the 70- to 79-year-old elderly age group (Pleyer et al. 2019).

Notably, congenital toxoplasmosis has been reduced in developed and developing countries. In the USA, NHANES surveys have shown a reduction of *T gondii* seropositivity in women of childbearing age from 31.7% in the late 1960s to 9.1% between 2009–2010 (Kimball et al. 1971, Jones et al. 2014). Similarly, in Colombia, the positivity rate of direct fluorescent antibody tests in samples from pregnant women decreased from 63.3% in 1993 to 45.7% in 2003, as measured by Microparticle Enzyme Immunoassay (Cañón-Franco et al. 2014). The reduction in congenital cases may be attributed to improved screening and treatment protocols during pregnancy and increased awareness of preventive measures (McAuley et al. 1994, Brézin et al. 2003). Consequently, the proportion of OT attributable to postnatally acquired infections is likely increasing worldwide (Gilbert 2000, Cifuentes-González et al. 2022).

## Age as a risk factor for ocular involvement after recent infection

The relationship between age and the risk of acquiring *T. gondii* infection and developing ocular involvement is complex and appears to vary throughout an individual’s lifetime. Due to risky behavior activities, such as geophagia, children can be exposed more to *T. gondii* infection, even those without congenital infections (Stagno et al. 1980). Contrary to what might be assumed from the lower frequency of observed active toxoplasmic retinochoroiditis episodes in older populations (Friedmann and Knox 1969, Gilbert et al. 1999, Bosch-Driessen et al. 2002), age does not necessarily reduce the risk of ocular involvement following a recently acquired *T. gondii* infection.

Studies in North America and Europe have shown that patients with OT who had serologic evidence of a recent infection were, on average, significantly older than those whose infections were acquired earlier in life (Ongkosuwito et al. 1999, Bosch-Driessen et al. 2002). Specifically, the mean age of patients with recently acquired infections presenting with ocular lesions was 50.6 years, compared to 29.9 years for those with older infections (Bosch-Driessen et al. 2002). Furthermore, primary retinal lesions, not associated with pre-existing scars, were more common in older patients, reinforcing that age is a critical risk factor (Ongkosuwito et al. 1999, Bosch-Driessen et al. 2002).

However, when considering clinical criteria for distinguishing between primary manifestations and recurrences, the age difference is not always consistently observed. Taghavi Eraghi et al. showed that the mean age for primary OT was 38.2 years, and for recurrent cases, 36.3 years (Eraghi et al. 2024).

In Southern Brazil, where *T. gondii* infection is endemic, the prevalence of OT increases with age. Studies in this region have consistently shown that older age groups are more likely to develop ocular disease following infection. For instance, Portela et al. found that OT was significantly higher among individuals older than 50 (Portela et al. 2004). In addition, Cifuentes et al., in a nationwide study in Colombia, documented a higher proportion of incident cases of OT in people over 50 (Cifuentes-González et al. 2022).

## Clinical characteristics according to age

Age is a critical factor influencing the severity and manifestations of OT. Traditionally, macular lesions have been considered indicative of congenital form. Specifically, Mets et al. (1996) demonstrated that 58% of newborns with congenital *T. gondii* infection had macular lesions, far exceeding what would be expected if lesions were distributed randomly, given that the macula comprises only ~5% of the retinal area (Mets et al. 1996). This disproportionate involvement of the macula has been corroborated by other studies, suggesting that early infection during retinal development may predispose this area to damage (Friedmann and Knox 1969, Dodds et al. 2008). Anatomical and microvascular differences between the macula and peripheral retina, such as the lower presence of macrophages in the macula, have been proposed to create a microenvironment more susceptible to lesion formation (Yang et al. 2000).

High rates of early retinochoroidal involvement (80%) and active lesions (50%) have been reported in Brazil in patients with congenital toxoplasmosis; this may be attributed to higher parasite virulence and individual susceptibility (Vasconcelos-Santos et al. 2009).

Clinical studies in adults with acquired *T. gondii* infection have also demonstrated high rates of macular involvement, with 55% reported in a Congolese cohort and 73% in a Colombian population (de-la-Torre et al. 2009, Nsiangani-Lusambo et al. 2023). These findings suggest that while age may influence lesion location, other factors such as host immune responses, particularly in immunocompromised individuals, and the virulence of *T. gondii* strains also play important roles in the manifestation and severity of the disease (de-la-Torre et al. 2013, Pfaff et al. 2014, Kalogeropoulos et al. 2022). The severity of OT is also notably higher in older patients. Studies focusing on older populations have reported that many patients present with severe disease characterized by multiple active lesions, large lesions greater than three-disc areas (DA), and prolonged disease duration exceeding 8 weeks (Johnson et al. 1997, Labalette et al. 2002). In these cases, IgM antibodies were reported in 41% of patients, suggesting recent infection rather than a cumulative effect of recurrent disease over many years.

Additional evidence suggests that older patients experience more severe inflammation and larger lesion sizes than younger individuals. In an international multicenter study, patients over 60 were significantly more likely to have lesions >1 DA in size than younger patients (77% vs. 42%, *P* = 0.020) (Dodds et al. 2008). Moreover, severe vitreous inflammation was more common in older patients, indicating a possible decline in immune function with age that limits the body’s ability to control the parasite effectively.

These assumptions of age being a significant factor influencing disease severity were further analyzed in a retrospective study of 290 OT patients. Older patients exhibited larger retinal lesions, with both variables measured metrically (R2 = 0,32, *P* < 0.005), and showed higher inflammatory activity in the vitreous (39.4 years vs. 30.4 years, *P* < 0.005) and anterior chamber (41.9 years vs. 34 years, *P* < 0.005). The aqueous humor antibody index (AI), which indicates the intraocular immune response, was significantly lower in older individuals (mean < 35 years: 45.1 ± 82.7 vs. ≥35 years: 18.6 ± 50.5, *P* = 0.046), suggesting a reduced capacity to control the infection locally. Consequently, older patients had lower baseline visual acuity (*P* = 0.043) and poorer visual outcomes after treatment (*P* = 0.019). In this study, complications such as macular and peripapillary edema and retinal detachment were not correlated with age but were more strongly associated with lesion location (*P* < 0.005) (Eraghi et al. 2024). These results highlight the impact of immunosenescence on disease severity in older patients. As the immune system ages, its ability to effectively respond diminishes, leading to more extensive retinal damage and prolonged disease courses. This underscores the importance of prophylactic therapy in elderly patients with OT, as they are at higher risk for poor outcomes (Reich and Mackensen 2015).

## Age and ocular toxoplasmosis recurrences

The inability of current treatment strategies to eliminate bradyzoites in the retina increases the risk of recurrence. A cluster pattern was first described in a Netherlands population where recurrence is highest immediately after an active episode and diminishes as the disease-free interval lengthens (Holland et al. 2008). Further research validates this pattern in the Colombian population (de-la-Torre et al. 2009). The patient’s age also influences the risk of recurrence at the first and subsequent episodes. Specifically, the relative risk of recurrence decreased by 15% for each decade increase in age at the first episode. Still, patients older than 40 years at the time of an active episode were at a higher risk of recurrence compared to younger individuals (Holland et al. 2008). This increased risk in older patients may be due to a decline in immune surveillance, which reduces the body’s ability to control parasites reactivating from tissue cysts (Weiskopf et al. 2009). However, other studies support the idea that younger patients also have a high risk of recurrence. For instance, a study in Switzerland found that recurrences were significantly more common in patients under 30 years of age than in older individuals (Garweg et al. 2008). This paradox can be explained by the complex interaction between age at first infection and subsequent recurrences. While older age at the time of an active episode is associated with an increased risk of recurrence, this risk diminishes over time, reflecting a protective effect associated with longer intervals since the initial infection. In the Netherlands cohort, the recurrence risk declined by 72% with each decade since the first episode, indicating that time since infection plays a crucial role in recurrence risk (Holland et al. 2008).

## Impact of age and ocular immune response

Infection with *T. gondii* generally generates an innate and adaptive immune response. Upon infection, the parasite triggers several pro-inflammatory cytokines, especially interferon gamma and IL-12, which enable the proliferation of the pathogen (Mantilla-Muriel et al. 2020, Miyagaki et al. 2024). T lymphocytes, especially CD 4+T cells, play a significant role in mediating this response by producing cytokines that activate macrophages and other immune cells to eliminate the parasite (Kalogeropoulos et al. 2022). However, the immune reaction, which is sometimes excessive, also causes tissue damage, which can be functionally decisive, especially in the eye (Miyagaki et al. 2024). Age significantly influences this immune response. In elderly patients, the immune system is characterized by immunosenescence, affecting innate and adaptive immune responses. This leads to reduced infection control and, thus, a higher risk of pronounced ocular damage. As a result, older patients often present with atypical findings due to more severe ocular toxoplasmosis, and the course of the infection is less favorable as compared to younger individuals. Recent studies further corroborate these findings. De Angelis RE et al. reported that older age was associated with more extensive lesions (>1 DA, *P* = 0.047) (De Angelis et al. 2021)

## Influence of age and ocular toxoplasmosis treatment

Despite advances in understanding the host–parasite interaction, there is currently no definitive method to eliminate *T. gondii* from ocular infections. Existing treatments, such as antibiotics and corticosteroids, can only control active infections and reduce inflammation, but they do not eradicate the parasite. Treatment for OT is commonly based on a combination of antibiotics and corticosteroids to control the parasite’s replication and manage inflammation. Several antibiotics have been used. Classically, triple therapy, including pyrimethamine plus sulfadiazine paired with folinic acid to mitigate side effects, has been implemented. Treatment patterns have shifted in recent years, and Cotrimoxazole and Clindamycin have become more common (Yogeswaran et al. 2023, Eraghi et al. 2024).

On the one hand, this is based on their better tolerability, and, on the other hand, secondary prophylaxis using, e.g. long-term low-dose Cotrimoxazole, is increasingly being carried out to reduce the risk of recurrence. Corticosteroids like prednisolone reduce inflammation and prevent damage to ocular tissues, always combined with antibiotic/antiparasitic therapy. The outcomes of these treatments generally include the resolution of active inflammation and improvement in visual acuity, although some patients may experience recurrences. Age plays a significant role in the clinical presentation and treatment outcomes of ocular toxoplasmosis. Older patients tend to have more severe disease manifestations, larger retinal lesions, and poorer visual outcomes compared to younger individuals. Also, the tolerability of certain antibiotics must be age-adapted, particularly for the youngest and oldest patients affected by OT (Yogeswaran et al. 2023, Eraghi et al. 2024).

## Conclusion

**Figure 1.**
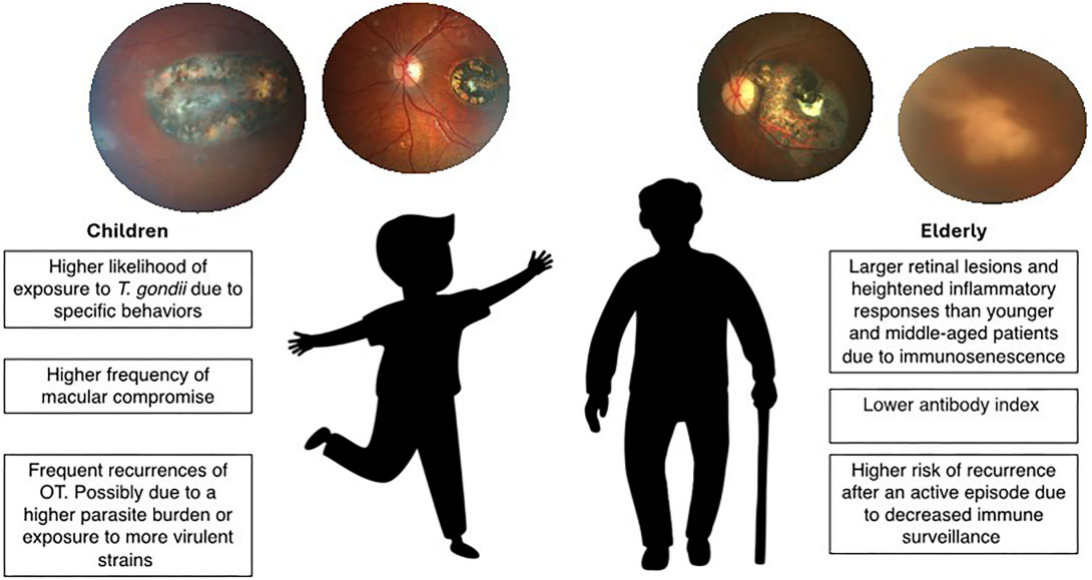
Key age-related differences in ocular toxoplasmosis (OT: Ocular toxoplasmosis).

This study underscores the significant influence of age on the epidemiology, clinical characteristics, and recurrence patterns of OT (Fig. 1). The relationship between age and the disease course is complex, involving immunological changes across the lifespan that affect the severity and manifestation of OT. Younger patients tend to exhibit more frequent macular involvement, while older individuals, due to immunosenescence, often experience more extensive lesions and severe inflammation. Furthermore, the risk of recurrence is intricately linked to the age at first infection, with older patients facing a higher likelihood of subsequent episodes. These findings highlight the importance of age-specific management strategies in optimizing outcomes for patients with OT.

## Supplementary Material

xtaf002_Supplemental_Files
