# Supplementary material for: Age and ocular toxoplasmosis: a narrative review
Source: FEMS Microbes. 2025 Feb 26;6:xtaf002. doi: 10.1093/femsmc/xtaf002 (PMC11912558; doi:10.1093/femsmc/xtaf002)
Supplement: xtaf002_Supplemental_Files [file xtaf002_supplemental_files.zip › FEMSMC-2024-038.R1 one sentence summary.docx]

Age is a critical determinant in the pathogenesis, clinical features, and outcomes of ocular Toxoplasmosis, with different age groups exhibiting distinct immunological responses and disease manifestations.
