# Supplementary material for: Age and ocular toxoplasmosis: a narrative review
Source: FEMS Microbes. 2025 Feb 26;6:xtaf002. doi: 10.1093/femsmc/xtaf002 (PMC11912558; doi:10.1093/femsmc/xtaf002)
Supplement: xtaf002_Supplemental_Files [file xtaf002_supplemental_files.zip › FEMSMC-2024-038.R1 supplementary data - reviews and author response.docx]

**Reviews of manuscript FEMSMC-2024-038**

**Reviewer 1: Heloisa Nascimento, Federal University of Sao Paulo**

**Recommendation: Accept**

congratulations

**Reviewer 2**

**Recommendation: Major Revision**

In the review manuscript entitled "Age and Ocular Toxoplasmosis: A Narrative Review" the authors summarize this important topic in an authoritative manner. The cited references are mostly up-to-date. However, the overall presentation and thus readability for non-experts could be significantly improved, as suggested below.

Major points

1. The abstract is missing! Please provide.

2. While this is a contribution to a thematic issue, other readers of the journal would very much appreciate a short paragraph introducing Toxoplasma gondii (its basic biology and medical characteristics). Currently, one has to have this prior knowledge to be able to follow. Please provide.

3. Along those lines: I was expecting some discussion about treatment options, their outcomes and whether they are influenced by age (and how).

4. The same for immune responses in the eye and how they are affected by age. It lights up sometimes, but a concise chapter addressing both points would be beneficial.

Minor points

5. page 3 line 36: a discussion of similar recent data from The Netherlands (Van den Berg, 2023) should be included here as well.

6. line 38: citation is missing (it is not Wilking, I guess).

7. line 51 ff: there is a recent study that should be discussed here as well (10.3201/eid3005.231045)

8. page 4, line 11. Quote" Similarly, in Colombia, positive direct fluorescent antibody tests were reduced in samples of pregnant women from 63.3% in 1993 to 45.7% positive Microparticle Enzyme Immunoassay in 2003". I know what is meant, but the tests were not reduced - rather the values. Please rephrase.

9. same page. I couldn't find Stagno et al 1980

10. page 4 line 43 ff. Please provide a reference

11. page 5 line43: explain 1 DA

12. page 7: the reference styles are inconsistent (some journal names abbreviated, some not)

13. Several small typos throughout the text. Please correct.

**Editor Decision: Major Revision**

Please provide a point-by-point response to the criticism by reviewer 2.

**Author's Response to Decision Letter for (FEMSMC-2024-038)**

Included as separate document entitled ‘submitted__Response to reviewers _Toxo_Age_(1).docx (v1.0)’

**Reviews of manuscript FEMSMC-2024-038.r1**

**Reviewer 2**

**Recommendation: Accept**

All changes were addressed satisfactorily

**Editor Decision: Accept**
