# Supplementary material for: Age and ocular toxoplasmosis: a narrative review
Source: FEMS Microbes. 2025 Feb 26;6:xtaf002. doi: 10.1093/femsmc/xtaf002 (PMC11912558; doi:10.1093/femsmc/xtaf002)
Supplement: xtaf002_Supplemental_Files [file xtaf002_supplemental_files.zip › submitted__Response to reviewers _Toxo_Age_(1).docx]

December 4, 2024

Dear Editor in Chief and Reviewers,

Thank you for the comments and suggestions to FEMSMC-2024-038, a manuscript entitled “Age and Ocular Toxoplasmosis: A Narrative Review.” We have attentively read the Editorial office and reviewers’ comments and suggestions and amended them in the manuscript.

We would like to express our sincere gratitude for the insight and constructive feedback provided on our manuscript. Your expertise and thoughtful analysis have been invaluable to us.

Reviewer: 1

Comments to the Author

congratulations

- Answer: Thank you for your comment, we truly appreciate your review.

Reviewer: 2

Comments to the Author

In the review manuscript entitled "Age and Ocular Toxoplasmosis: A Narrative Review" the authors summarize this important topic in an authoritative manner. The cited references are mostly up-to-date. However, the overall presentation and thus readability for non-experts could be significantly improved, as suggested below.

Major points

1. The abstract is missing! Please provide.

- **Answer:** We added an abstract accordingly. “Toxoplasma gondii is an extremely “successful” opportunistic parasite that infects most warm-blooded animals, including humans. While the infection is generally largely asymptomatic, the infection of the eye presenting as ocular toxoplasmosis (OT) is a potentially blinding consequence. OT remains the most common cause of infectious retinochoroiditis and places a considerable socio-economic burden on societies, particularly in developing countries. Age is one of several factors influencing the clinical presentation and outcomes of OT. Older patients often exhibit more severe disease manifestations, larger retinal lesions, and poorer visual outcomes compared to younger individuals. This disparity is attributed to immunosenescence, the age-related decline in immune function, which impairs the body's ability to control the infection effectively. Consequently, older individuals are at a higher risk of severe complications and recurrent episodes of inflammation. Understanding the interplay between age and immune response is essential for developing targeted treatment strategies and improving patient outcomes in OT”. (Page 2; lines 1-15)

2. While this is a contribution to a thematic issue, other readers of the journal would very much appreciate a short paragraph introducing Toxoplasma gondii (its basic biology and medical characteristics). Currently, one has to have this prior knowledge to be able to follow. Please provide.

- **Answer:** We appreciate the reviewer’s suggestion to include a brief introduction to *Toxoplasma gondii*. To address this, we propose adding the following paragraph to the introduction section to ensure all readers have the necessary context

Toxoplasma gondii (T. gondii) is a versatile intracellular parasite that infects most warm-blooded animals, including humans. The parasite has a complex life cycle involving two main stages: tachyzoites, which rapidly multiply and spread the infection, and bradyzoites, which form tissue cysts and remain dormant. Felines are the definitive host where the parasite undergoes sexual reproduction. T. gondii infection can occur by ingestion of food or water contaminated with cysts or oocysts. Medically, toxoplasmosis remains often asymptomatic in healthy individuals but can cause flu-like symptoms such as fever, swollen lymph nodes, and muscle pain. Notable, ocular involvement is an important consequence of T. gondii infection… (Page 3; lines 3-15)

3. Along those lines: I was expecting some discussion about treatment options, their outcomes and whether they are influenced by age (and how).

- Answer: We appreciate the reviewer’s comment regarding the inclusion of a discussion on treatment options, their outcomes, and the potential influence of age. We propose adding the following text to the discussion section to address this point:

Despite advances in the understanding of the host-parasite interaction, there is currently no definitive method to eliminate T. gondii from ocular infections. Existing treatments, such as antibiotics and corticosteroids, can only control active infections and reduce inflammation, but they do not eradicate the parasite entirely. Treatment for ocular toxoplasmosis is commonly based on a combination of antibiotics and corticosteroids to control the replication of the parasite and to manage inflammation. Several antibiotics have been used. Classically, triple therapy including pyrimethamine plus sulfadiazine paired with folinic acid to mitigate side effects, has been implemented. In recent years, treatment pattern has shifted and Cotrimoxazole and Clindamycin became more common. (Yogeswaran et al. 2023; Taghavi-Eraghi, Garweg and Pleyer 2023) On the one hand, this is based on their better tolerability, and, on the other hand, secondary prophylaxis using e.g. long-term low dose Cotrimoxazole is increasingly being carried out to reduce the risk of recurrence. Corticosteroids like prednisolone are used to reduce inflammation and prevent damage to ocular tissues. The outcomes of these treatments generally include the resolution of active inflammation and improvement in visual acuity, although some patients may experience recurrences. Age plays a significant role in the clinical presentation and treatment outcomes of ocular toxoplasmosis. Older patients tend to have more severe disease manifestations, larger retinal lesions, and poorer visual outcomes compared to younger individuals. Also, tolerability of certain antibiotics must be age adapted, in particular for the youngest and oldest patients affected by OT (page 1; Lines 1-23)

4. The same for immune responses in the eye and how they are affected by age. It lights up sometimes, but a concise chapter addressing both points would be beneficial.

- Answer: We thank the reviewer for highlighting the need for a dedicated discussion on immune responses in the eye and their age-related variations. To address this, we propose including a concise section in the discussion that comprehensively covers these aspects:

Infection with T. gondii generally generates an innate and adaptive immune response. Upon infection, the parasite triggers several pro-inflammatory cytokines, especially interferon gamma and IL-12, which enable the proliferation of the pathogen (Mantilla-Muriel et al. 2020; Miyagaki et al. 2024). T lymphocytes, especially CD 4+T cells, play a significant role in mediating this response by producing cytokines that activate macrophages and other immune cells to eliminate the parasite (Kalogeropoulos et al. 2022). However, the immune reaction, which is sometimes excessive, also causes tissue damage, which can be functionally decisive, especially in the eye (Miyagaki et al. 2024). Age significantly influences this immune response. In elderly patients, the immune system is characterized by immunosenescence, affecting innate and adaptive immune responses. This leads to reduced infection control and, thus, a higher risk of pronounced ocular damage. As a result, older patients often present with atypical findings due to more severe ocular toxoplasmosis, and the course of the infection is less favorable as compared to younger individuals. Recent studies further corroborate these findings. De Angelis RE et al. reported that older age was associated with more extensive lesions (> 1 DA, p=0.047) (De Angelis et al. 2021) (Page 9; Lines 18-23)

Minor points

5. page 3 line 36: a discussion of similar recent data from The Netherlands (Van den Berg, 2023) should be included here as well.

- **Answer:** Thank you for providing this reference. We have now included a discussion of the Netherlands study, specifically highlighting the observed increase in seroprevalence among older patients. We added the following: "For example, in the Netherlands, the third nationally representative serosurvey on *T. gondii* seroprevalence conducted between 2016 and 2017 found an increase from 10.9% in children and adolescents aged 0–19 years to 36.7% in adults aged 20–89 years" (Page 5; lines 9-11).

6. line 38: citation is missing (it is not Wilking, I guess).

- **Answer:** Dear reviewer, thank you for noticing this; we corrected the reference to align with the journal guidelines (Krings et al. 2021) (Page 5; line 18).

7. line 51 ff: there is a recent study that should be discussed here as well (10.3201/eid3005.231045)

- **Answer:** Dear reviewer, thank you for Thasnk ting this reference. We have added a discussion of the study to the manuscript. Specifically, we included the following: "This rapid increase in seroprevalence during childhood has also been observed in Germany, where Giese et al. documented a T. gondii seroprevalence of 6.3% in female children and adolescents. With each additional year of life, the likelihood of being seropositive increased by 1.2, indicating a strong force of infection. Social status and municipality size were associated with seropositivity" (Page 5; lines 24-28).

8. page 4, line 11. Quote" Similarly, in Colombia, positive direct fluorescent antibody tests were reduced in samples of pregnant women from 63.3% in 1993 to 45.7% positive Microparticle Enzyme Immunoassay in 2003". I know what is meant, but the tests were not reduced - rather the values. Please rephrase.

- **Answer:** Dear reviewer, thank you for your observation. We have revised the sentence to clarify that it was the positivity rates that decreased, not the tests themselves. The revised sentence now reads: "Similarly, in Colombia, the positivity rate of direct fluorescent antibody tests in samples from pregnant women decreased from 63.3% in 1993 to 45.7% in 2003, as measured by Microparticle Enzyme Immunoassay" (Page 6; lines 4-6).

9. same page. I couldn't find Stagno et al 1980

- **Answer:** We apologize for not including the reference earlier in the manuscript. We have now added Stagno et al. 1980 as appropriate. The full reference is: Stagno S, Dykes AC, Amos CS et al. An outbreak of toxoplasmosis linked to cats. *Pediatrics* 1980;65:706–12.

10. page 4, line 43 ff. Please provide a reference

- **Answer:** Thank you for noticing this we add the reference (Eraghi, Garweg and Pleyer 2024) as suggested

11. page 5 line43: explain 1 DA

- **Answer:** Dear reviewer, "DA" was previously explained in the manuscript as referring to "disc areas," specifically in the context of "large lesions greater than three-disc areas (DA). (Page 7; line 34). No changes have been made to this section.

12. page 7: the reference styles are inconsistent (some journal names abbreviated, some not)

- **Answer:** We apologize for the inconsistency in the reference styles. We have modified the references, ensuring all journal names are now correctly abbreviated according to the standard formats.

13. Several small typos throughout the text. Please correct.

- Answer: We appreciate the reviewer’s observation regarding typographical errors. A thorough review of the manuscript has been conducted, and all identified typos have been corrected. Thank you for bringing this to our attention.
